# Establishment of an indirect ELISA detection method for porcine reproductive and respiratory syndrome virus NSP4

**DOI:** 10.3389/fmicb.2025.1549008

**Published:** 2025-02-18

**Authors:** Mengmeng Zhao, Chen Lv, Jiankun Pang, Zhiyu Yang, Huiyang Sha

**Affiliations:** Guangdong Provincial Key Laboratory of Animal Molecular Design and Precise Breeding, School of Animal Science and Technology, Foshan University, Foshan, China

**Keywords:** ELISA, NSP4, porcine reproductive and respiratory syndrome virus, prokaryotic expression, PRRs

## Abstract

The non-structural protein 4 (NSP4) of porcine reproductive and respiratory syndrome virus (PRRSV) is equipped with 3C-like serine protease (3CLSP) activity, influencing crucial aspects such as virus replication, host IFN-*β* suppression, host cell apoptosis induction, and PRRSV detection facilitation. In response to wild or attenuated PRRSV strains, antibodies against non-structural proteins are generated, while inactivated vaccines fail to elicit such responses. Employing the Enzyme-Linked Immunosorbent Assay (ELISA) method targeting non-structural proteins helps discern the immune effects of inactivated versus wild or attenuated vaccine strains. The study focused on the NSP4 protein from the PRRSV XH-GD strain (GenBank No. EU624117.1), which was cloned, expressed, and leveraged as a coating protein for establishing an indirect enzyme-linked immunosorbent assay (ELISA) detection method. This method showcased outstanding specificity, repeatability, and sensitivity, exhibiting a notable agreement rate of 91.74% with the PRRSV IDEXX ELISA kit. The successful development of the NSP4 indirect ELISA not only supports the detection of PRRSV antibodies but also provides a robust platform for ongoing antibody monitoring in pig farming. Utilizing PRRSV NSP4 for ELISA antibody detection offers a more sustainable approach for continuous surveillance. The high agreement between this method and commercial kits lays a solid groundwork for effectively differentiating between inactivated and attenuated vaccines, enhancing the management and monitoring of PRRSV in pig populations.

## 1 Introduction

Porcine reproductive and respiratory syndrome virus (PRRSV) induces fever and anorexia in infected pigs. Late-stage pregnant sows may experience miscarriages, premature births, and stillbirths, while neonates are particularly vulnerable to respiratory complications. Highly pathogenic strains cause symptoms like severe fever, cyanosis, and sudden death in adult pigs (Sha et al., 2022). After PRRSV infection, the virus undermines herd immunity and increases susceptibility to co-infections. Its persistence hampers the complete eradication of the virus and poses significant challenges to China’s livestock industry (Lunney et al., 2010).

PRRSV non-structural proteins (NSPs) play crucial roles in viral replication and expression. NSP4 is derived from the polyproteins pp1a and pp1ab to serve as a replicase and transcriptase during the processing of NSP3–NSP12, facilitating the assembly and maturation of viral particles (Allende et al., 1999; van Dinten et al., 1999). NSP4 possesses two canonical *β*-barrel trypsin-like structures and a conserved C-terminal domain of undetermined function (Dokland, 2010). The NSP4 gene is highly conserved and anti-NSP4 antibodies are detectable during the early phases of PRRSV infection (Tian et al., 2009). The enzymatic function of the NSP4 3C-like serine protease (3CLSP) is critical for modulating PRRSV replication, attenuating host interferon responses, and precipitating host cell apoptosis, underscoring its significance as a pivotal target for elucidating the pathogenic mechanisms underlying PRRSV (Aeksiri and Jantafong, 2017; Liu et al., 2020; Wei et al., 2020). Numerous viruses, including PRRSV, modulate interferon production upon host interaction to circumvent the host’s innate immune defenses. Specifically, PRRSV NSP4 impedes type I interferon (IFN-I) production and has been implicated in multiple immunosuppressive activities that can critically undermine the host’s innate immune response. In addition to counteracting the IFN-I signaling pathway, NSP4 activates the Notch signaling pathway, modulates antigen presentation, facilitates cell apoptosis, and plays additional roles in the evasion of host defenses. NSP4 is predominantly synthesized during viral replication, making it a promising diagnostic antigen for the detection and differentiation between natural infections and responses to immunization with inactivated vaccines (Qi et al., 2017; Zhang et al., 2017; Zhao et al., 2007).

In this study, specific primers targeting the conserved region of PRRSV NSP4 were designed. The NSP4 gene from PRRSV XH-GD was amplified by polymerase chain reaction (PCR) and expressed in the vector, pET-28a-NSP4 to produce purified NSP4 to use as a coating antigen. The conditions for indirect enzyme-linked immunosorbent assay (ELISA) to detect PRRSV antibodies were optimized and the specificity, sensitivity, and repeatability of the method were rigorously evaluated, which was then compared to that of the PRRSV IDEXX ELISA kit. The overarching goal was to formulate an indirect ELISA for PRRSV NSP4 with heightened specificity, sensitivity, and robustness to mitigate the financial challenges associated with PRRSV monitoring and diagnosis.

## 2 Materials and methods

### 2.1 Virus, bacterial, and serum samples

The pET-28a (+) plasmid, *Escherichia coli* (*E. coli*) DH5α (Vazyme Biotech Co., Ltd., Nanjing, China), *E. coli* BL21 (DE3) (Vazyme Biotech Co., Ltd., Nanjing, China), and PRRSV XH-GD strain (GenBank No. EU624117.1) were maintained in the laboratory. Seventy-two PRRSV-positive and 158 PRRSV-negative serum samples were stored and validated using the PRRSV IDEXX ELISA kit. The laboratory-preserved serum samples were positive for swine Japanese encephalitis virus (JEV), porcine circovirus type 2 (PCV2), porcine epidemic diarrhea virus (PEDV), porcine pseudorabies virus (PRV), and African swine fever virus (ASFV). All samples are stored at −20°C before use.

### 2.2 Target gene amplification and cloning

Using the NSP4 gene sequence of the PRRSV XH-GD strain from NCBI (GenBank No.EU624117.1) as a reference, primers were designed with Primer Premier 5 software to incorporate *Hind* III (Takara Bio Inc., Dalian, China), and *Xho* I (Takara Bio Inc., Dalian, China) restriction enzyme sites: NSP4-F: 5′- GAGAAGCTTGCGGCGCTTTCAGAACT-3′ (*Hind* III site underlined); NSP4-R: 5′- CCGCTCGAGATTCCAGTTCGGGTTT-3′ (*Xho* I site underlined), and primer protective sites, restriction sites and anti-migratory sites were also considered in the design. The primers were synthesized by Tianyi Huiyuan Biotechnology Co., Ltd. Using cDNA synthesized from the PRRSV XH-GD strain and the specified primers, the NSP4 fragment was PCR-amplified using 2 × Phanta Flash Master Mix 2 (Vazyme Biotech Co., Ltd., Nanjing, China), the PCR reaction system was 2 × Phanta Master 25.0 μL, 2.0 μL each of NSP4-F and NSP4-R, 1.0 ng of plasmid, and 50.0 μL of supplementation using ddH_2_O. The PCR regimen included an initial denaturation at 95°C for 3 min, 35 cycles at 95°C for 10 s, 55°C for 30 s, and 72°C for 60 s, and a final extension at 72°C for 10 min. The resulting PCR products were stored at −20°C. After dual digestion of both the PCR product and pET-28a plasmid with *Xho* I and *Hind* III, the target fragment and vector were isolated and ligated using T4 DNA ligase (New England Biolabs Ltd., Beijing, China) according to the manufacturer’s protocol. The mixture was transformed into competent *E. coli* DH5α and candidate positive clones were selected on Luria-Bertani (LB) agar plates supplemented with 50 μg/mL kanamycin. Following expanded growth, the recombinant plasmid was extracted and verified by double digestion with *Xho* I and *Hind* III. The correctly sequenced recombinant plasmids were designated as pET-28a-NSP4 after sequencing validation by Tianyi Huiyuan Biotechnology Co., Ltd.

### 2.3 Target gene expression, purification, and validation

Both the recombinant plasmid pET-28a-NSP4 and the empty pET-28a vector, were introduced into *E. coli* BL21 (DE3). Positive transformants were isolated on LB agar plates supplemented with 50 μg/mL kanamycin. Selected colonies were inoculated into LB broth supplemented with 50 μg/mL kanamycin and incubated overnight at 37°C to establish seed cultures. The cultures were then expanded at a 1:100 dilution until they reached an OD600 nm of approximately 0.6. Isopropyl *β*-D-1-thiogalactoside (IPTG) (Beyotime Biotech Inc., Shanghai, China) was added to a final concentration of 1.0 mmol/L to instigate NSP4 expression. At intervals spanning 1 to 7 h post-induction, 1 mL aliquots were collected, centrifuged at 12,000 rpm for 1 min, and bacterial pellets were harvested for protein extraction and subsequent sodium dodecyl sulfate-polyacrylamide gel electrophoresis (SDS-PAGE) (Solarbio Co., Ltd., Beijing, China) analysis. Iterative optimizations were conducted for IPTG dosage, induction duration, and temperature to determine the optimal NSP4 expression parameters. Using the established conditions, the induced bacterial culture was centrifuged to collect the cells, which were then lysed with sonication. After centrifugation, both the supernatant and the pellet were subjected to SDS-PAGE to ascertain the predominant expression modality of NSP4. The recombinant protein was subsequently expressed and purified under denaturing conditions using a His-tagged protein purification kit (denaturant-resistant) (Beyotime Biotech Inc., Shanghai, China). The integrity of the purified protein was confirmed SDS-PAGE and Coomassie blue staining. The concentration of the purified protein was determined using a BCA protein quantification kit (Vazyme Biotech Co., Ltd., Nanjing, China).

### 2.4 Western blotting

The recombinant plasmid pET-28a-NSP4 and empty pET-28a vector were transformed into *E. coli* BL21 (DE3) for protein induction. Following protein induction, the samples were subjected to 12% SDS-PAGE to evaluate protein expression. After electrophoresis, the proteins were transferred onto a polyvinylidene fluoride (PVDF) membrane (Beyotime Biotech Inc., Shanghai, China) placed within a transfer cassette oriented in the following order: sponge pads, a tri-layer of pre-soaked filter paper positioned on the cathode side, followed by the gel, a PVDF membrane (pre-activated with methanol for 3 min), and another tri-layer of pre-soaked filter paper and sponge pad. The roller tool was carefully passed over the assembly to ensure that no air bubbles were trapped between the layers. The constructed sandwich was integrated into the transfer apparatus and immersed in an ice bath to maintain a low temperature during the process. To ensure proper orientation, with the PVDF membrane facing the anode, electrotransfer was performed at a constant voltage of 80 V for 1 h.

Following electrophoretic transfer, the PVDF membrane was removed and submerged in 5% skim milk solution (Beyotime Biotech Inc., Shanghai, China) for 1 h at room temperature. After blocking, the membranes were washed twice with Tris-buffered saline containing Tween-20 (TBST) (Solarbio Co., Ltd., Beijing, China) and gently agitated on a horizontal shaker for 10 min per wash. The primary antibody was prepared by diluting PRRSV-positive serum (1:1000) and His-tag (10E2) mAb (1:1000) (Abmart Co., Ltd., Shanghai, China), anti-*β*-actin (Solarbio, Beijing, China, # K200058M, WB (1:1000)) in a solution containing 5% bovine serum albumin (BSA) (Labgic Co., Ltd., Anhui, China). The membrane was incubated with the antibody dilution and overnight at 4°C. The secondary horseradish peroxidase (HRP)-labeled goat anti-pig IgG/HRP (1,5,000) (Solarbio Co., Ltd., Beijing, China) or HRP-conjugated anti-mouse IgG binding protein (Santa Cruz Biotechnology, #sc-525409, dilution 1:50000) were diluted in 5% skim milk solution. The PVDF membrane was then immersed in secondary antibody and incubated for 1 h at room temperature. Then, the membrane was washed three times with TBST for 10 min each with gentle agitation on a rocking platform. For signal visualization, the membrane was treated with a chemiluminescent substrate using an ultrasensitive ECL chemiluminescence kit (Beyotime Biotech Inc., Shanghai, China) and luminescent emissions were recorded using imaging equipment (Tanon Co., Ltd., Shanghai, China).

### 2.5 Optimization of antigen coating concentration and serum dilution

The purified recombinant NSP4 antigen was subjected to sequential dilution at concentrations ranging from 0.25 μg/mL to 8.00 μg/mL using ELISA coating buffer (Solarbio Co., Ltd., Beijing, China). A 100 μL volume of each antigen concentration was pipetted into the wells of an ELISA plate and incubated overnight at 4°C. After incubation, the excess antigen was washed five times with PBST (Solarbio Co., Ltd., Beijing, China) for 5 min each. For blocking, 200 μL/well 3% BSA solutions were added and the plate was incubated at 37°C for 3 h before the plate was washed five times with PBST. Standard serum samples were initially diluted at a 1:50 ratio and further serially diluted to 1:1600. Serum samples (100 μL/well) were added to the ELISA plate and incubated at 37°C for 1 h to develop the foundation for the ELISA matrix experiments. After another washing step, 100 μL/well goat anti-pig IgG/HRP (Solarbio Co., Ltd., Beijing, China) diluted at 1:10000 was added and the plate was incubated at 37°C for 1 h. The plate was washed again and 100 μL/well TMB single-component substrate solution (Solarbio Co., Ltd., Beijing, China) was added for 15 min at 37°C. The chromogenic reaction was terminated by adding 50 μL/well ELISA stop solution (Solarbio Co., Ltd., Beijing, China). Optical densities were assessed at OD_450_ nm (OD_450_) to determine the optimal conditions for antigen coating and serum dilution.

### 2.6 Optimization of ELISA experimental conditions

The best protein coating was applied to ELISA plate, followed by a series of blocking solutions. These solutions included 1% BSA, 3% BSA, 5% BSA, 1% skim milk, 3% skim milk, 5% skim milk, 1% fetal bovine serum (FBS), 3% FBS, and 5% FBS, which were used based on the initial determination of the optimal coating protein concentration and serum dilution. To determine the optimal incubation time for the primary antibody, intervals of 15, 30, 45, 60, 75, and 90 min were methodically assessed under consistent experimental conditions. Subsequently, goat anti-pig IgG/HRP was serially diluted at 1:5000, 1:10000, 1:15000, and 1:20000. For precision in the secondary antibody reaction, incubation times of 15, 30, 45, 60, 75, and 90 min were evaluated. Finally, the optimal color development time was assessed at 5, 8, 10, 15, and 20 min.

### 2.7 Critical value of ELISA method

For the established ELISA method, 40 porcine serum samples previously identified as PRRSV antibody-negative were tested and confirmed with the PRRSV IDEXX test kit (IDEXX Laboratories, Inc.,). The mean and standard deviation (SD) of the OD_450_ values of the negative serum samples were calculated. The ELISA positive/negative threshold was determined by comparing the mean ± 3 SD of theOD_450_ values from the negative serum samples.

### 2.8 Evaluation of assay specificity, sensitivity, and reproducibility

Under optimized conditions, assessments of JEV, PCV2, PEDV, PRV, and ASFV were conducted to evaluate potential cross-reactivity and specificity of the established ELISA detection method. A comprehensive serial dilution of 12 levels: 1:10, 1:20, 1:40, 1:80, 1:160, 1:320, 1:640, 1:1280, 1:2560, 1:5120, 1:10,240, and 1:20,480 was used for the PRRSV standard positive serum. Using the established indirect ELISA antibody detection method, the maximum serum dilution factor was determined based on the test results to provide insights into the sensitivity of the detection method. The precision of the method was evaluated using both intra- and inter-batch assessments. Using the methodology developed in this study, eight randomly selected pig serum samples were tested on the ELISA plates coated with the same batch of antigens. Each sample underwent three repeated measurements and the results were subjected to statistical analysis, including the calculation of coefficients of variation, to facilitate the observation of changes in intra-batch reproducibility. Furthermore, using the methodology established in this study, another set of eight randomly chosen pig serum samples was tested using ELISA plates coated with antigens from different batches, which were statistically analyzed with coefficients of variation to illustrate any changes in inter-batch reproducibility.

### 2.9 Comparative analysis of the indirect ELISA with the PRRSV IDEXX ELISA kit

Two hundred and thirty clinical porcine serum samples were concurrently analyzed using the indirect NSP4 ELISA method established in this study and the PRRSV ELISA IDEXX kit. The results were analyzed and the conformity rate was calculated.

## 3 Results

### 3.1 Cloning results of the recombinant plasmid pET-28a-NSP4

**Figure 1:**
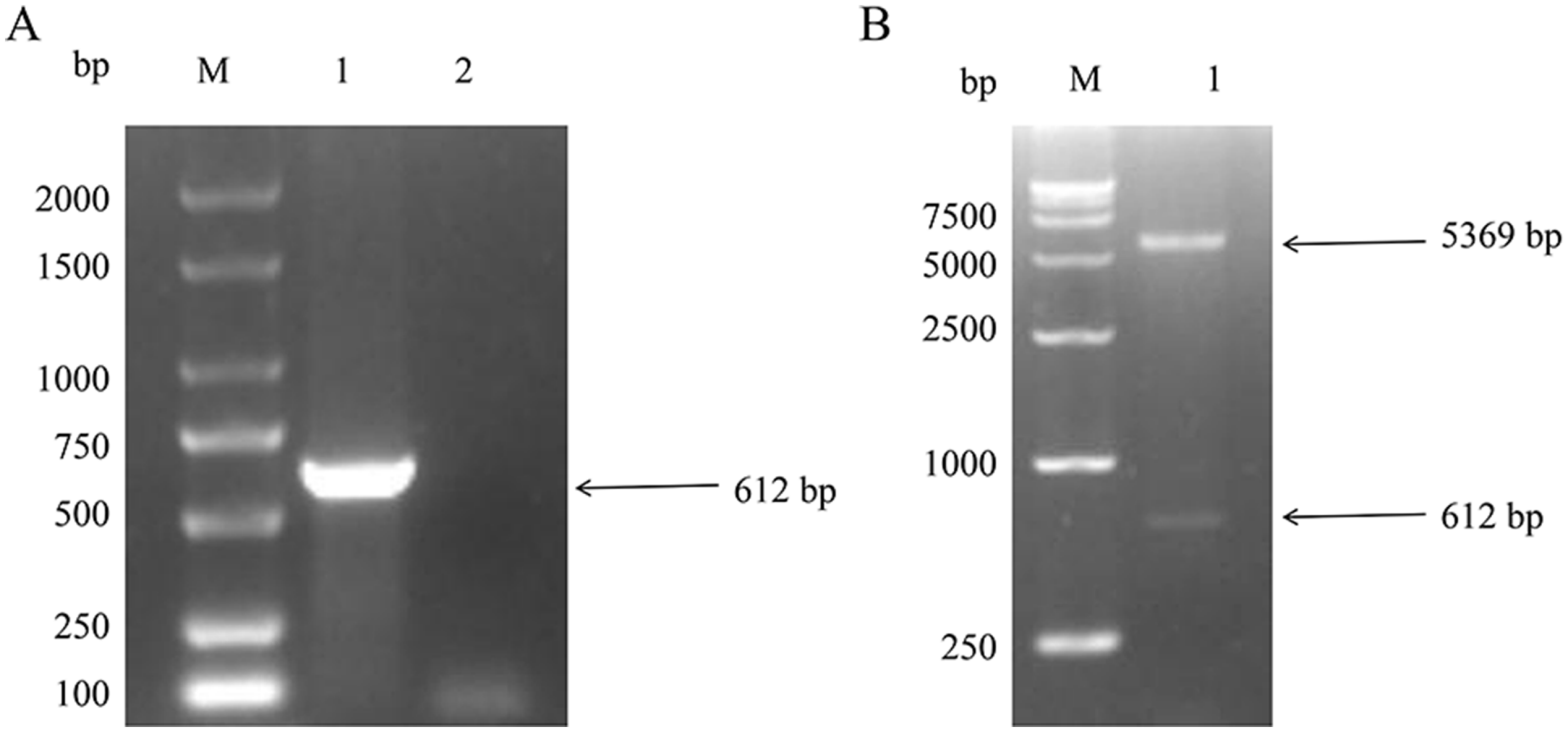
Construction of pET-28a-NSP4 vector. **(A)** PCR amplification result of the NSP4 gene fragment from the PRRSV XH-GD strain. M. DL2000 plus DNA marker (Vazyme, #MD101), PCR products for 1. NSP4 and 2. the negative control. **(B)** Identification of the double-enzyme digestion product of the recombinant plasmid, pET-28a-NSP4. M. DL15000 DNA marker (Vazyme, #MD103) and 1. the double-enzyme digestion product of the pET-28a-NSP4 plasmid.

The PRRSV NSP4 gene was amplified by PCR from the XH-GD strain (Figure 1A). The amplified NSP4 was then directionally ligated into the prokaryotic expression vector pET-28a to create the recombinant expression vector, pET-28a-NSP4. The integrity and correct insertion of NSP4 into the vector was confirmed by a double digestion assay using the restriction enzymes *Hind* III and *Xho* I (Figure 1B). To further validate the construction, double-digested pET-28a-NSP4 was sequenced and the resulting sequence alignment was congruent with the expected insert. Collectively, these experimental findings confirmed the successful generation of the recombinant prokaryotic expression vector, pET-28a-NSP4.

### 3.2 Induction and validation of NSP4 protein expression

**Figure 2:**
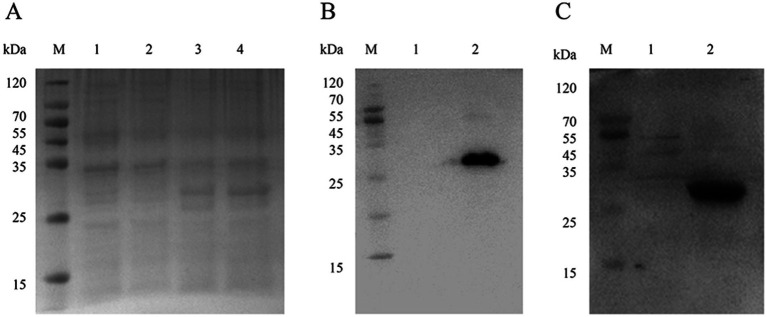
Expression of pET-28a-NSP4 vector in *Escherichia coli.* **(A)** Identification of the recombinant NSP4 protein by SDS-PAGE. M. 15–120 KDa protein marker, 1. pET-28a empty vector without IPTG induction, 2. pET-28a empty vector with 1 mM IPTG induction for 5 h, 3. pET-28a-NSP4 without IPTG induction, and 4. pET-28a-NSP4 with 1 mM IPTG induction for 5 h. **(B)** Identification of NSP4 recombinant protein using western blotting with an anti-His monoclonal antibody. M. 15–120 KDa protein marker, 1. purified pET-28a-NSP4 recombinant protein, and 2. IPTG induced empty vector control. **(C)** Identification of NSP4 recombinant protein using western blotting with positive serum. M. 15–120 KDa protein marker, 1. purified pET-28a-NSP4 recombinant protein, and 2. IPTG induced empty vector control.

The recombinant pET-28a-NSP4 construct was introduced into the prokaryotic expression host *E. coli* BL21 (DE3). Upon induction with IPTG, the expression of the NSP4 recombinant protein was observed as a discernible protein band that corresponded to the anticipated molecular weight of 27 KDa was produced by SDS-PAGE. In contrast, the sample harboring the empty pET-28a vector displayed no such bands, confirming the selective expression of NSP4 (Figure 2A).

Through rigorous optimization of the expression parameters, we found that the most efficacious induction time was 7 h at 37°C with 0.8 mmol/L IPTG. After induction, bacterial cultures were harvested, sonicated, and centrifuged such that the supernatant could be separated from the cell pellet. SDS-PAGE analysis of these fractions revealed the presence of the NSP4 protein in the pellet, indicating that the NSP4 recombinant protein manifested predominantly as inclusion bodies. Following the solubilization of these inclusion bodies using urea, the protein was purified using affinity chromatography on a nickel column and used in western blotting analyses with both His-tag (10E2) mAb and PRRSV-positive serum to validate the identity and integrity of the purified NSP4 protein. The blots demonstrated not only recognition of the recombinant protein by the His-tag (10E2) mAb (Figure 2B) but also its specific reactivity with PRRSV-positive serum (Figure 2C). These results underscored the reactivity and authenticity of the expressed NSP4 protein.

### 3.3 Optimization of PRRSV NSP4 indirect ELISA reaction conditions

**Figure 3:**
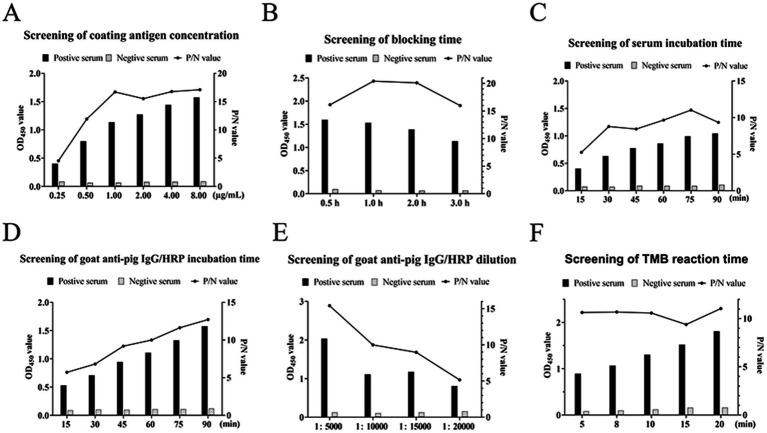
Optimization of ELISA reaction conditions. Screening for the optimal **(A)** coat antigen concentrations, **(B)** blocking time, **(C)** serum incubation time, **(D)** goat anti-pig IgG/HRP incubation time, **(E)** goat anti-pig IgG/HRP dilution, and **(F)** TMB reaction time. The left axis indicates the absorbance (OD450) and the right axis indicates the ratio of the positive control to the negative control (P/N value).

Purified recombinant NSP4 served as the antigenic foundation for formulating an indirect ELISA tailored for PRRSV antibody detection. The optimization of the operational conditions resulted in the establishment of pivotal parameters. The optimal NSP4 protein coating concentration was ascertained at 1.00 μg/mL using a checkerboard titration method and a corresponding serum dilution set at 1:400. The preferred blocking buffer composition included 3% BSA with a 3 h incubation. Serum reactions were conducted at 37°C for precisely 75 min. The optimal goat anti-pig IgG/HRP dilution was 1:10000, with a 1 h incubation, while the optimal TMB single-component substrate solution coloration incubation was 8 min (Figure 3). The development of the indirect ELISA for NSP4 antibody detection was predicated using a systematic exploration of the aforementioned optimal conditions. Using this analytical methodology, a cohort of 40 clinical porcine serum samples previously identified as PRRSV-negative using the PRRSV IDEXX ELISA kit and archived in our laboratory were analyzed using the indirect PRRSV NSP4 ELISA method. The mean OD_450_ reading for 40 negative serum samples was 0.190, with a SD of 0.052. Therefore, the threshold for delineating positive and negative results was 0.346.

### 3.4 Evaluation of ELISA assay specificity, sensitivity, and reproducibility

**Figure 4:**
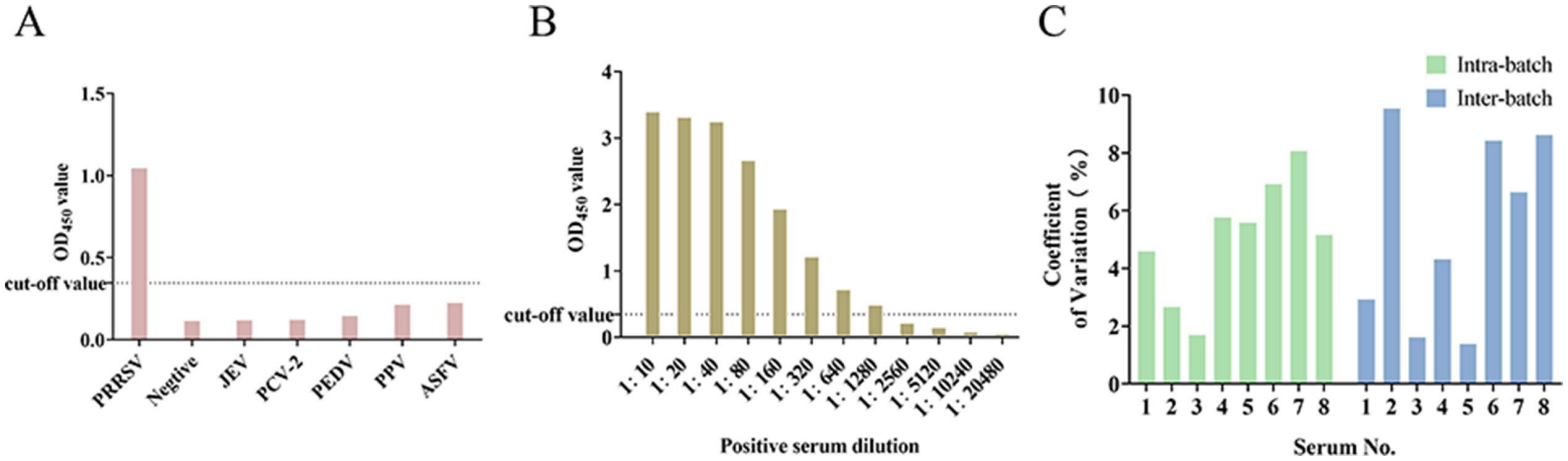
Results of specificity, sensitivity, and coefficient of variation of the detection method. **(A)** Specificity test of the ELISA assay. **(B)** Sensitivity test of the ELISA assay. **(C)** Coefficient of variation of intra-batch and inter-batch tests.

When exposed to the sera positive for JEV, PCV2, PEDV, PRV, and ASFV, the observed OD450 values for all positively-identified serum antibodies were consistently below the established cutoff threshold. This indicated that the sera did not exhibit cross-reactivity with the established NSP4 indirect ELISA detection method, demonstrating the robust specificity of the assay (Figure 4A). The serial dilution of standard positive serum (1:10–1:20,480) demonstrated that even at a dilution of 1:1280, the OD450 values remained above the critical threshold, confirming the method’s robust sensitivity (Figure 4B). Inter- and intra-batch reproducibility tests were conducted using the established NSP4 indirect ELISA detection method. Reproducibility analysis indicated that the coefficients of variation for both the intra-batch and batch-to-batch replicate experiments were consistently below 10%, indicating excellent repeatability of the method (Figure 4C).

### 3.5 Comparative analysis with PRRSV IDEXX ELISA kit

A set of 230 porcine serum samples was examined using both the developed indirect NSP4 ELISA and the commercially available PRRSV IDEXX ELISA kit. The results from the two methodologies revealed that the developed assay identified 75 samples as positive, while the PRRSV IDEXX ELISA kit flagged 72 samples as positive. Notably, there was an overlap in the 64 samples that were positively identified by both assays, with simultaneous identification of all 147 negative samples within the cohort, resulting in a concordance rate of 91.74% (Table 1).

**Table 1:** Comparison of IDEXX and NSP4 ELISA tests.

Detection method		Results of detection using the IDEXX ELISA commercial kit		
		Positive No.	Negative No.	Total No.
Results of PRRSV NSP4 indirect ELISA detection method	Positive No.	64	11	75
	Negative No.	8	147	155
	Total No.	72	158	230
Coincidence rate		88.89%	93.04%	91.74%

## 4 Discussion

In this study, the recombinant PRRSV NSP4 protein was successfully expressed and protein purification was performed under denaturing conditions to obtain a homogeneous protein devoid of impurities. Given the inherent sequence conservation of NSP4 (Sha et al., 2023), the designed NSP4 indirect ELISA promises versatility for the diagnosis of a variety of PRRSV strains. Western blotting results suggest that the bacterially-expressed NSP4 protein reacts well with PRRSV-positive serum, highlighting the potential reactivity of recombinant NSP4. The tests for specificity, sensitivity, and reproducibility produced consistent results and when we compared our NSP4 indirect ELISA with the established PRRSV IDEXX ELISA kit using 150 clinical serum samples, we observed a high concordance rate, underscoring the reliability of the new method.

A timely and efficient PRRSV detection strategy is paramount for proactive management and mitigation of PRRSV outbreaks. Several techniques have been refined and widely adopted for PRRSV detection, including laboratory-based methodologies for virus isolation (VI), ELISA, indirect immunofluorescence assay (IFA), immunoperoxidase monolayer assay (IPMA), PCR, quantitative real-time PCR (qPCR), digital PCR (dPCR), loop-mediated isothermal amplification (LAMP), recombinase polymerase amplification (RPA), clustered regularly interspaced short palindromic repeats (CRISPR), and metagenomic next-generation sequencing (mNGS) (Mazziotta et al., 2024; Pan et al., 2023; Terzapulo et al., 2024). Serological assays characterized by their throughput capabilities are important tools in epidemiological surveillance and comprehensive diagnostic (Costa et al., 2024; Leme et al., 2017). Serological diagnostic techniques are predominantly used because they are straightforward, cost-effective, and require limited specialized equipment (Qiu et al., 2021). ELISA is a flexible and precise serological modality that is indispensable for monitoring the prevalence of PRRSV and evaluating vaccine efficacy. ELISA offers the dual advantage of streamlining the detection workflow and having diverse assay sensitivity and specificity. Various ELISA kits have been developed for PRRSV antibody detection. Assessing antibody levels remains the gold standard for assessing vaccine-induced immunological responses, highlighting the importance of devising effective vaccination strategies. The commercial PRRSV ELISA kids include the IDEXX HerdChek PRRS Ab X3 (IDEXX Laboratories, Inc., Westbrook, ME, USA; hereafter abbreviated as IDEXX-ELISA) and LSIV ET Porcine PRRS/AS-Serum (Laboratoire Service International, Lissieu, France; abbreviated as LSI-ELISA). In a comparative analysis, Ge et al. evaluated the merits of these kits in the context of the HP-PRRSV vaccination regimen and reported that IDEXX-ELISA outperformed LSI-ELISA for early antibody detection, thus presenting a more viable option for preliminary HP-PRRSV infection diagnosis (Ge et al., 2019).

PRRSV infection remains a significant challenge in the Chinese swine industry and effective serological monitoring is crucial for identifying and managing PRRSV-positive swine farms. The predominant method for detecting PRRSV antibodies is the IDEXX-ELISA, which has precise antibody detection. However, a significant limitation is the inability to discriminate between antibodies elicited by the wild-type virus and those produced in response to vaccination. Hence, screening for a suitable antigen to detect antibodies against the wild-type PRRSV is a focus of serological research on PRRSV. NSP4 is an integral replicase and transcriptase component that is required for modulating PRRSV replication, inhibiting IFN, inducing apoptosis, and facilitating early diagnosis of infection. Its inherent ability to stimulate antibody production makes NSP4 an exemplary antigen for the identification of wild-type PRRSV antibodies. This study used the pET prokaryotic expression system to express the NSP4 protein, which showed pronounced protein expression levels, was cost-effective, and retained NSP4 reactivity, which ensured it would not compromise subsequent detection techniques. Proteins derived from pET vectors have multiple applications ranging from protein activity research and mutant characterization to reagent preparation and structural studies. Although many vectors are suitable for expressing analytical quantities of proteins for screening or antigenic purposes, a combination of the optimal vector, host bacterium, and cultivation conditions is required for scalable purification. Initially, our methodology incorporated the pET-32a vector but we encountered challenges with suboptimal protein expression. After iterative refinement, we transitioned to the pET-28a vector, which was more efficacious. The expressed fusion protein was characterized by a His-tag conFigureuration, facilitating its binding with nickel ions and the ease of protein purification. Ordinarily, pET vectors exhibit stability in *E. coli,* reducing concerns of plasmid attrition over extended periods. However, during this study, bacterial samples cultured from −80°C storage demonstrated no expression of the recombinant protein. This aberration indicated plasmid loss that potentially stemmed from inadequate selective pressure, unstable plasmid replication, or suboptimal cultivation parameters. To mitigate these challenges, it is important to maintain optimal antibiotic concentrations, appropriate storage conditions for plasmid-stabilized strains, and conducive culture conditions, as well as conduct regular bacterial passaging.

The utility of ELISA for detecting serum antibodies is supported by its high throughput, sensitivity, and operational simplicity, making it the mainstay of antibody quantification. The indirect ELISA methodology developed in this study harnesses the non-structural protein of PRRSV to discern antibodies from bona fide PRRSV infections. Consequently, on swine farms where inactivated PRRSV vaccines are administered, this method could help differentiate between viral- and vaccine-derived antibodies. This distinction in method application also sets the stage for discerning between inactivated and attenuated vaccine immunizations.

NSP4 is a pivotal player in the initial phases of viral replication and persists for an extended timeframe in the PRRSV replication cycle. NSP4-specific antibodies have be reported as early as 14 days post-inoculation with PRRSV-2 VR2332 and persist for up to 202 days. This prolonged antibody response makes NSP4 particularly beneficial for early diagnosis of PRRSV infections, and also be used for PRRSV long-term monitoring and tracking (Fang and Snijder, 2010). In addition, the NSP4 indirect ELISA method can help to differentiate between different types of vaccine immunisation, such as inactivated vaccine immunisation and immunisation with weakly virulent vaccines, thus guiding the development and adjustment of rational immunisation programmes.

Currently, the predominant market offerings include imported PRRSV antibody-testing kits that serve their purpose but are expensive and sometimes lack precision in detecting neutralizing antibody levels. In this study, an advanced indirect ELISA targeting the PRRSV NSP4 was established to address the challenges associated with the high cost of commercially available PRRSV antibody diagnostic kits and their analytical limitations. By refining the detection process, our approach guarantees accurate and dependable PRRSV antibody results, thereby improving foundational PRRSV antibody testing and monitoring. Our method also offers increased testing efficiency, which is suitable for comprehensive clinical sample analyses. When compared with conventional test kits, this method is a cost-effective diagnostic alternative for swine production, enabling the development of more precise preventive strategies against PRRSV outbreaks. Although the indirect ELISA for PRRSV NSP4 established in this study had a high concordance of results with the PRRSV IDEXX ELISA kit, this study has not yet been validated against an unknown large number of serum samples, and thus this study has some limitations. In summary, the NSP4 indirect ELISA detection method developed in this study has significant application value both to researchers and pig farming industries.

## 5 Conclusion

This study successfully expressed the recombinant PRRSV NSP4 protein with robust antigenicity and established an indirect ELISA detection method. Through comparison with PRRSV ELISA IDEXX kit, we validated the reliability and efficiency of this method, providing a novel option for PRRSV antibody detection and monitoring at the grassroots level. Additionally, we delved into the protein expression process and proposed optimized expression conditions for the pET vector, offering valuable insights for similar future studies. In summary, the PRRSV NSP4 indirect ELISA established in this study holds promising application prospects. It is expected to provide a more effective and cost-efficient means of detecting and controlling PRRSV, thereby playing a significant role in the development and health maintenance of the swine industry.

## Data Availability

The original contributions presented in the study are included in the article/Supplementary material, further inquiries can be directed to the corresponding author.
