# Supplementary material for: Establishment of an indirect ELISA detection method for porcine reproductive and respiratory syndrome virus NSP4
Source: Front Microbiol. 2025 Feb 18;16:1549008. doi: 10.3389/fmicb.2025.1549008 (PMC11876416; doi:10.3389/fmicb.2025.1549008)
Supplement: Supplementary file 2 [file Presentation_1.pptx]

## Slide 1
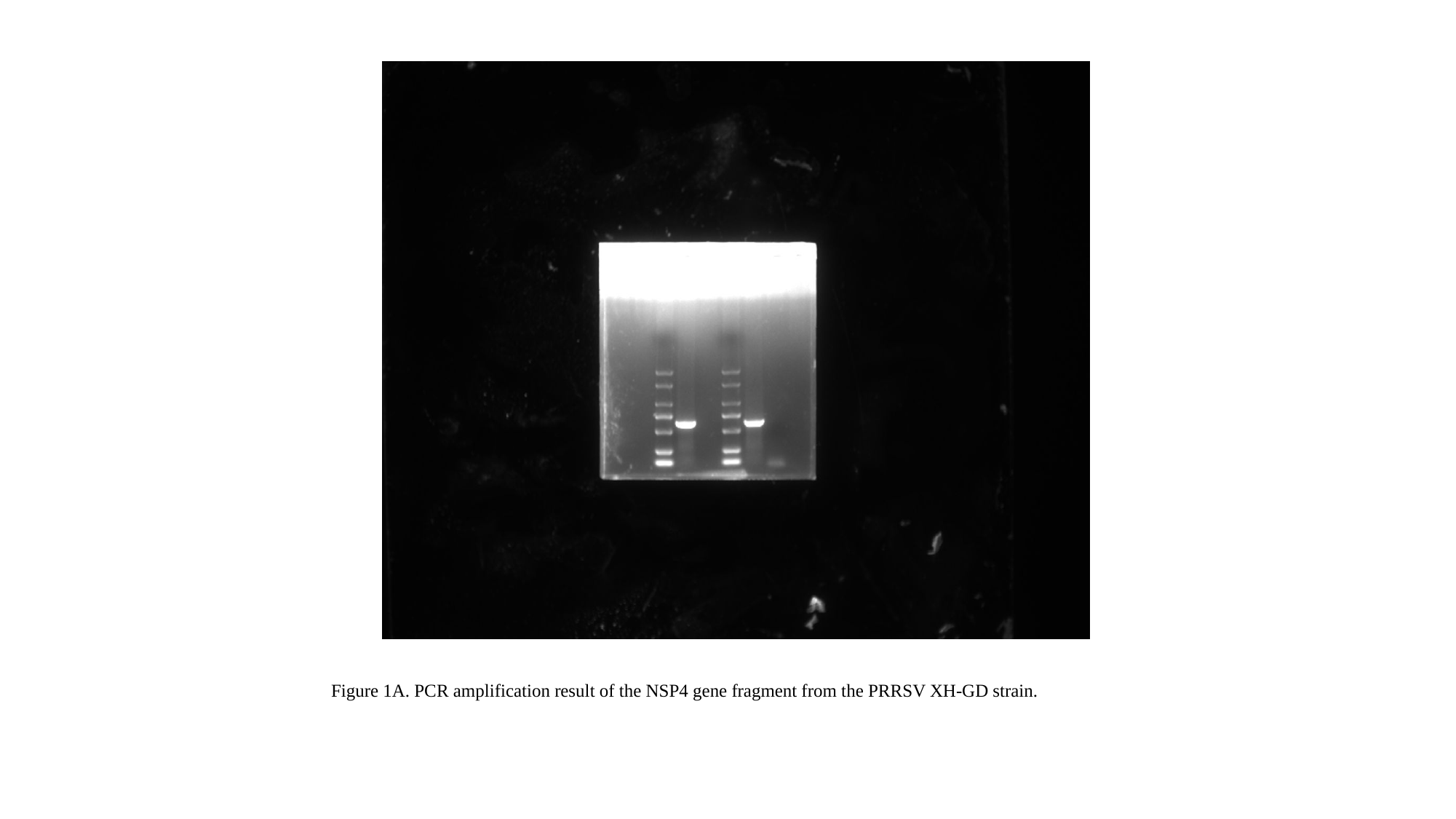

Figure 1A. PCR amplification result of the NSP4 gene fragment from the PRRSV XH-GD strain.

## Slide 2
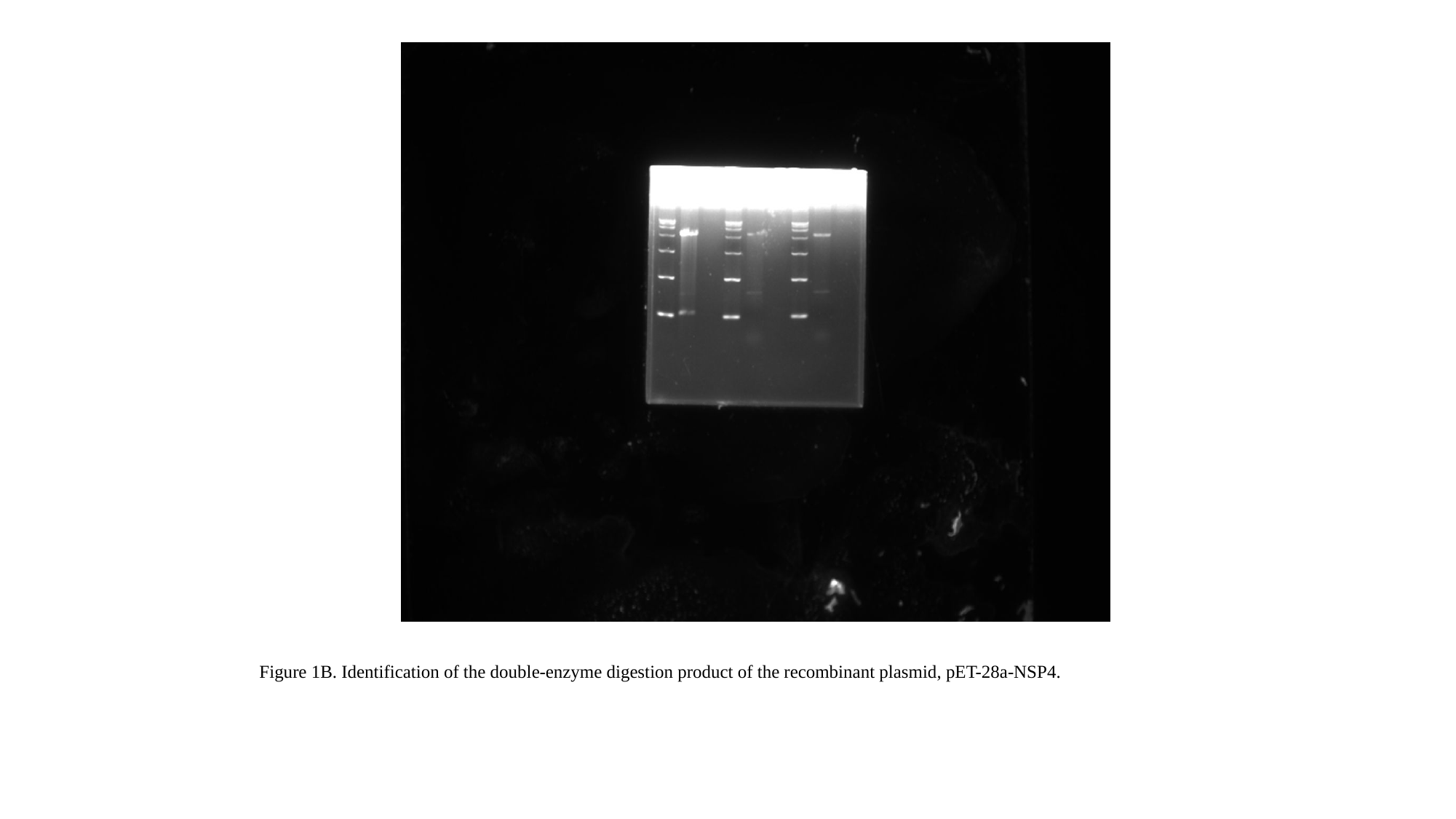

Figure 1B. Identification of the double-enzyme digestion product of the recombinant plasmid, pET-28a-NSP4.

## Slide 3
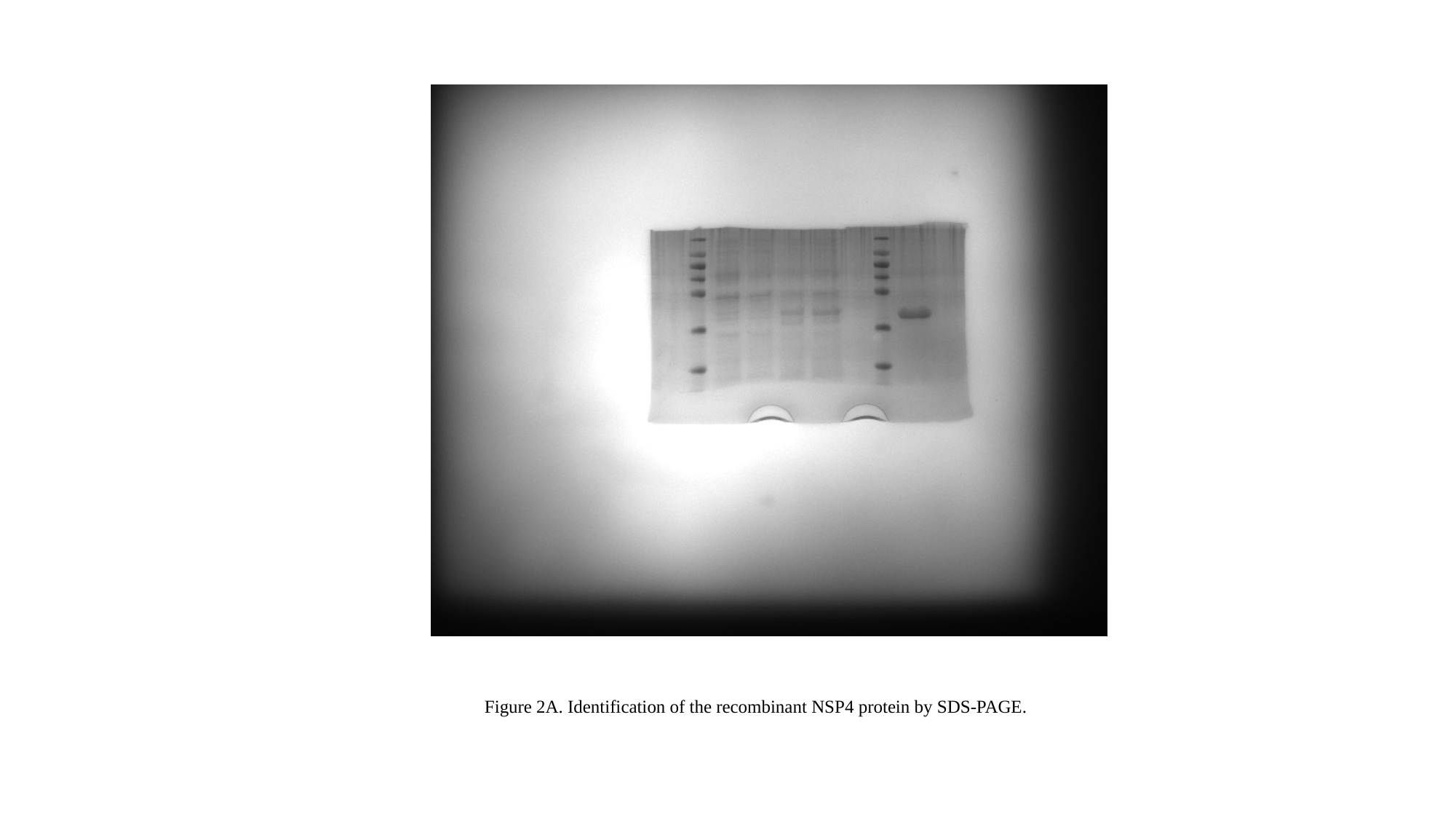

Figure 2A. Identification of the recombinant NSP4 protein by SDS-PAGE.

## Slide 4
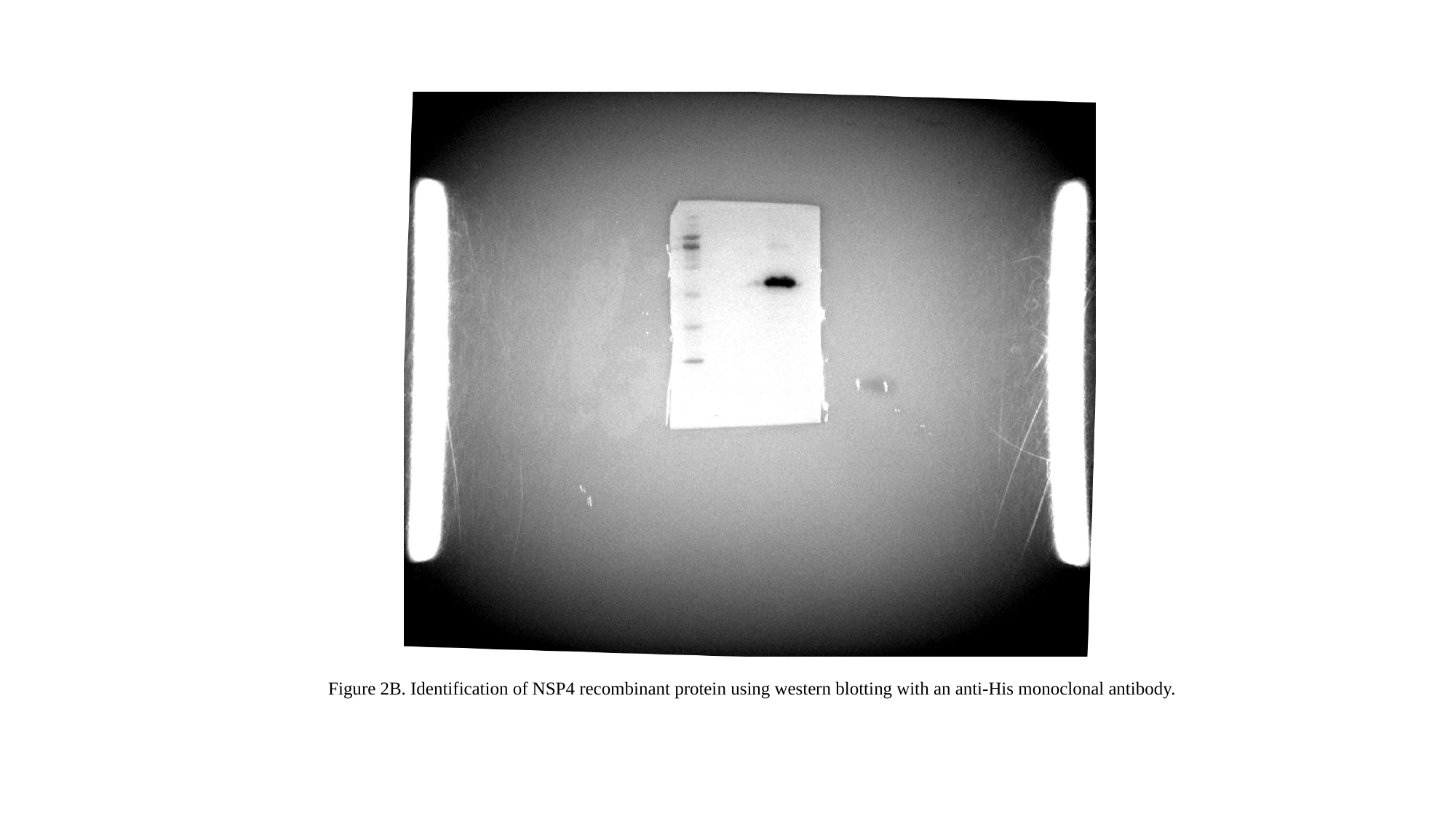

Figure 2B. Identification of NSP4 recombinant protein using western blotting with an anti-His monoclonal antibody.

## Slide 5
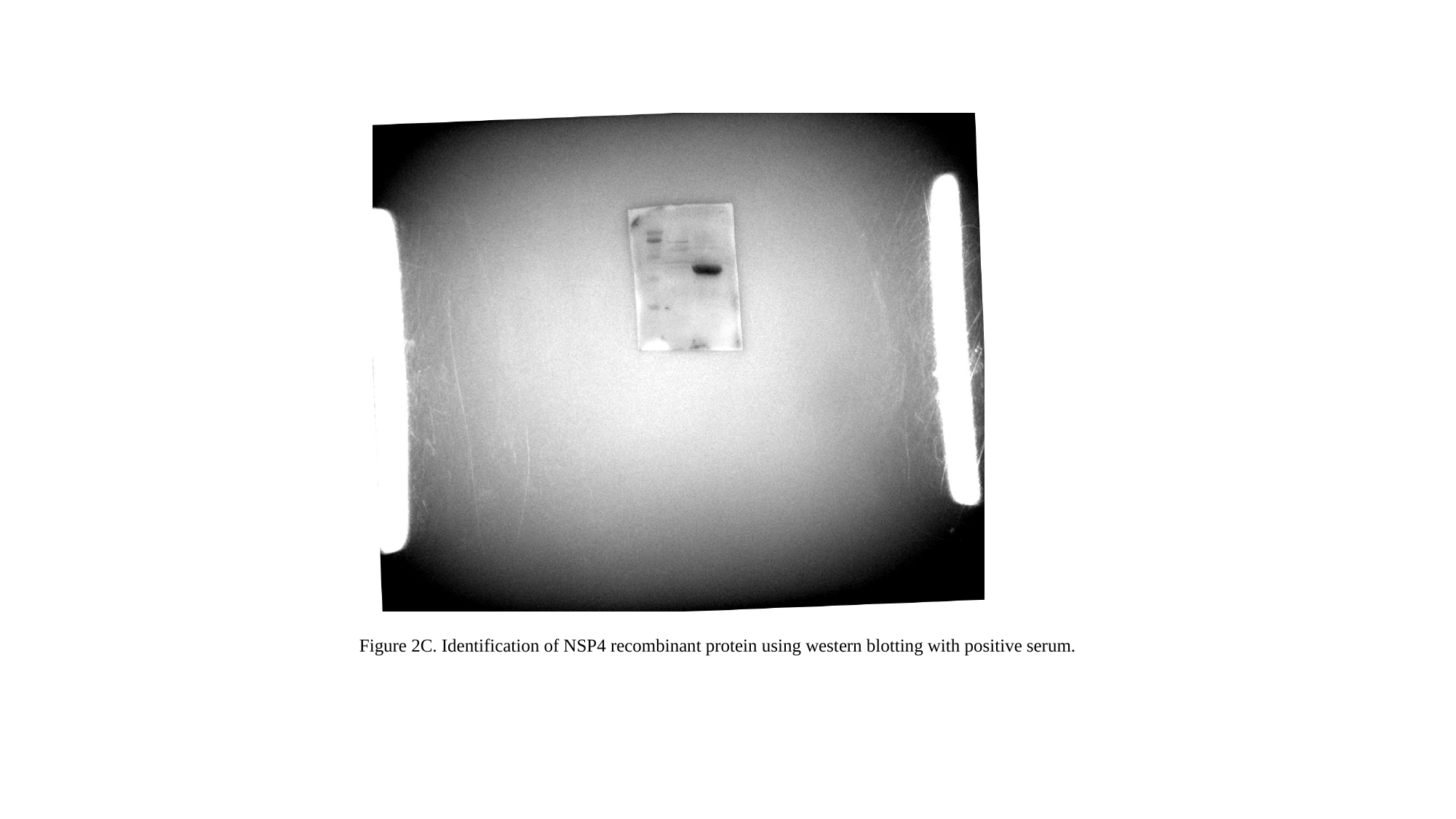

Figure 2C. Identification of NSP4 recombinant protein using western blotting with positive serum.
